# Time to first birth and its determinants among married female youths in Ethiopia, 2020: survival analysis based on EDHS 2016

**DOI:** 10.1186/s12905-021-01414-1

**Published:** 2021-08-02

**Authors:** Desalegn Anmut Bitew, Yohannes Ayanaw Habitu, Abebaw Addis Gelagay

**Affiliations:** Department of Reproductive Health, Institute of Public Health, College of Medicine and Health Sciences, University of Gondar, P.O. Box 196, Gondar, Ethiopia

**Keywords:** Time to first birth, Determinants, Married female youths, Ethiopia

## Abstract

**Introduction:**

The first birth is the most significant events in a woman's life that indicates the beginning of undertaking the intensive responsibilities of motherhood and childcare. Age at first birth has health, economic and social consequences and implications. But little has been known on the time to first birth and its determinants in Ethiopia. Therefore, this research is planned to address this issue.

**Objectives of the study:**

To assess the time to first birth and its determinants among married female youths in Ethiopia, 2020.

**Methods:**

The data was accessed freely through (https://www.dhsprogram.com). Survival analysis of time to first birth was done based on EDHS 2016 data among 2597 weighted study subjects. The data was extracted using STATA version 14.0. Kaplan Meier’s survival and Log rank test were used to compare survival experiences of respondents using categorical variables. Proportional hazard assumption was checked and was not violated. Cox proportional hazard model was applied, hazard ratio with 95% CI was computed and variables with *p* value < 0.05 in the multivariable analysis were taken as significant determinants.

**Results:**

Overall median survival time was 18 years (IQR = 17–20). The significant determinants of time to first birth are place of residence (being rural (AHR = 1.49, 95% CI 1.13, 1.97),Religion (being Muslim [AHR = 1.57, 95% CI 1.22, 2.02),being protestant (AHR = 1.73, 95% CI 1.34, 2.24)], age at first sex [first sex < 15 years (AHR = 1.68, 95% CI 1.23, 2.29)] and first sex between 15 and 17 years (AHR = 1.54, 95% CI 1.29, 1.85), age at first marriage (marriage < 15 years (AHR = 6.52, 95% CI 4.91, 8.64), marriage between 15 and 17 (AHR = 2.63, 95% CI 2.20, 3.14), unmet need for family planning (AHR = 1.23, 95% CI 1.00, 1.52)

**Conclusion:**

In this study, the median age at first birth was 18 years. This show, about 50% of study participants give birth for the first time before their 18th birth day. This age is the ideal age for schooling and to do other personal development activities. Therefore giving birth before 18 year will limit female youths from attending school and performing personal development activities in addition to health and demographic consequences of early child bearing.

## Introduction

The first birth is one of the most significant event in a woman’s life, which can affect the welfare of women, men, and children indicating the beginning of undertaking the intensive responsibilities of motherhood and childcare [1]. Girls in low-income countries face complex fertility and schooling decisions with the added constraints of low availability of information on safe sexual practices and limited access to reproductive health services [2].

Scholars say that the best time to get pregnant is between late 20 s and early 30 s. This age range is associated with the best outcomes for both the mother and her baby [4]. One study pointed that mothers optimal age at first birth, associated with the maximum expected health, is around 30.5 [5].Childbearing among adolescents and youths is a common sexual and reproductive health (SRH) issue among young people, particularly in developing countries [6].

Fertility patterns in the world have changed dramatically producing a world with very diverse child bearing patterns after the international conference on population and development (ICPD) in 1994, [7]. Sub-Saharan Africa is the only region in the world, where fertility decline has been slow and late [8]. Most countries in Sub Saharan Africa are still experiencing relatively higher fertility rates [9].According to United Nations 2014 report, out of 66 high fertility countries 45 (more than 3.2 children per woman) are concentrated in sub-Saharan Africa [7].

Nearly 16 million adolescent girls between 15 and 19 give birth each year which accounts roughly 11% of all births worldwide; 95% occur in developing countries [10].

Ethiopia is the second most populous country in Africa and is characterized by high population growth of 2.5% annually [11]. The fertility rate was around 5 children per woman, and most of the populations are young people. In 2016, nearly half (47%) of the total population was under 15 years old and 20% of the population is aged 15–24, of whom 47% are sexually active and 13% of women age 15–19 have begun childbearing [12].

Delaying pregnancy and child bearing among young women may contribute to higher school attainment, which might translate into better human capital outcomes for their children [3]. First birth determine women’s reproductive pattern and also affect countries fertility transition [13]. The economic and social consequences of youth’s pregnancy and early child bearing are sever in developing countries, which is associated with maternal mortality, low birth weight, low school attainment and productivity, and consequently intergenerational transmission of poverty [10, 14, 15]. Early maternal age at first birth and multiparty are associated with chronic diseases which may be associated with poor physical performance, osteoporosis, diabetes, chronic lung disease, high blood pressure, stroke, coronary heart disease, arthritis, and cancer [17–19], and have strong and unwelcome association with low levels of educational achievement for young women, which in turn may have a negative impact on their position and potential contribution to society [20]. Early first childbirth has been reported as the main cause of high population growth and high fertility, high maternal mortality and morbidity [15, 21–23].

Women’s fertility level is affected by different factors such as availability of effective birth control methods, age at marriage, age at first sexual intercourse, religious beliefs, acceptability of abortion, infant mortality rate, educational and career development opportunities, economic factors and urbanization [24–27].

Ethiopia has national adolescent and youth health strategy to address the sexual and reproductive related issues including preventing early child bearing [28] but early child bearing is still high about 13 births for every 100 young women aged 15–19 years [12]. Even if early child bearing has multiple adverse health, economic and demographic consequences, little has been known on the determinants of time to first birth among youth population in Ethiopia.

Few available literatures on this issue mainly focus on either teenagers or all reproductive women regardless of their marital status. The current study focuses on youths who were given little attention by the previous researchers and being married also enabled to address factors related to their husbands. Updated knowledge using nationally representative data could provide useful information for policy makers to implement future interventions for controlling the total fertility rate and reduce the health, demographic and socioeconomic consequences of first child bearing at early age.

Therefore, the objective of this research project was to determine time to first birth and to identify its determinants among ever married youth females.

## Methods

### Study setting, data source, population and period

The study was conducted in Ethiopia using the 2016 Ethiopian demographic and health survey data. In Ethiopia according to the EDHS 2016 report, the median age at first marriage and first sexual intercourse were 17.1 years and 16.6 years respectively, the maternal mortality ratio was 412 per 100,000 live births, skilled delivery coverage was 28%, the contraceptive prevalence among married women and a fertility rate was 36% and 4.46 respectively and Sexually active unmarried women accounts 58% [12]. The principal investigator requested Central Statistics Agency (CSA) to get access the data. The data was accessed freely through (https://www.dhsprogram.com). The data extraction period was from 1st June to 1st July. Weighted sample was 2597. About 2597 participants were involved after weighting.

### Variables of the study

The dependent variable in this study was the time (age in years) at first birth. Whereas the independent variables were Socio-demographic and economic factors (educational status, employment status, residence, religion and economic status), reproductive factors (number of miscarriages and stillbirths before first birth**,** use of contraceptives, age at first sex and age at first marriage), behavioral factors (cigarette smoking**,** chat chewing, alcohol drinking), media related factors (media exposure and hearing family planning messages on mass medias) and husband related factors (husband education, husband occupation, husbands desire for child and husband’s behavioral factors like alcohol drinking, chat chewing and cigarettes smoking).

### Operational definitions

*Event*: giving first birth.

*Censored*: Not giving first birth.

*Survival time*: The time is taken in years (age) from her birth to give her first birth.

### Sampling methods

**Fig. 1:**
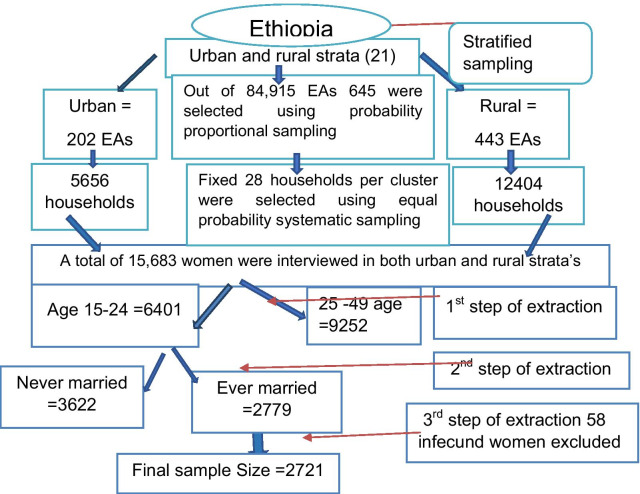
Schematic representation of the sampling procedures in the study of time to first birth and its determinants among married youth females in Ethiopia, 2016. The final sample size after being weighted by sampling weight was 2597. *N.B EAs* Enumeration Areas

A total of 2597 respondents were included in this study. All sampling procedures are available at the EDHS reports of each year. But to highlight the sampling procedures and step of data extraction we use the following diagram (Fig. 1).

### Data processing and analysis

Survival analysis was used to identify the determinants of age at first birth.

All procedures of Ethiopian Demographic and health survey were performed based on the international ethical guideline of the DHS program [12] and the data processing and analysis in this document were done based on a step-wise guide to perform survival analysis [30].

The data for this study were extracted from EDHS 2016 individual (women) record folder using STATAversion14software. The extracted data was recorded, cleaned using frequency; listing and sorting to identify any missed values. Kaplan Meier's survival was used to estimate the time of first birth and used to compare survival experiences among categories across time. A log-rank test was used to compare survival experiences between two or more groups and to show the statistical significance of the observed difference in the Kaplan Meier's survival plot. The PH assumption was checked both graphically and using global test. A global test based on Schoenfeld residuals found that all of the covariates and the full model satisfied the proportional hazard assumption (Chi square = 16.35 and *p* value = 0.0900). As stated above the data source for this study was the EDHS data which has hierarchical nature (that means youths in the same cluster may have similar characteristics than youths in another cluster). As a result, we have checked the presence or absence of between cluster variability/ heterogeneity by fitting frailty model (random effect Cox regression model) and the theta value at the null model was insignificant (*p* value > 0.05). It indicates that there is no unobserved heterogeneity between clusters. Multicollinearity was also checked and the maximum variance inflation factor (VIF) was 2.62 for age at first marriage and the mean VIF was 1.67. This shows that no multicollinearity between covariates. Variables which were associated with the outcome variable in the bivariable analysis at *p* value < 0.25 were entered into a multi-variable analysis. The cutoff point for the significant association was *p* value < 0.05 at 95% confidence interval.

### Rationale for use of survival analysis

Survival analysis is analysis of history of events which uses statistical procedures to analyze time duration, until one or more events of interest happen. Time-to-event (TTE) data is unique because the outcome of interest is not only whether or not an event occurred, but also when that event occurred. Logistic and linear regression is not suited to be able to include both the event and time aspects as the outcome in the model. These regression methods also are not equipped to handle censoring, a special type of missing data that occurs in time-to-event analyses when subjects do not experience the event of interest during the follow-up time [29].

## Results

### Socio-demographic distribution of the study participants

In this study the weighted sample size was 2597 ever married female youths. Among these respondents 2224 (85.6%) were from the rural residency. One thousand sixty four (41.0%) of the respondents were orthodox. When we see their economics status, 1157 (44.5%) of respondents were in the poor economic class. Eight hundred seventy-three (33.6%) of respondents have no formal education (Table 1).

**Table 1:** Socio-demographic distribution of married female youths in Ethiopia, 2016

Variables	Categories	Weighted frequency	Weighted percentage
Residence	Urban	373	14.4
	Rural	2224	85.6
Religion	orthodox	1064	41.0
	Muslim	970	37.3
	Protestant	486	18.7
	Others^a^	78	3 .0
Educational status of respondents	No education	873	33.6
	Primary	1314	50.6
	Secondary	304	11.7
	Higher	106	4.1
Wealth index	Poor	1157	44. 5
	Middle	521	20.1
	Rich	919	35.4
Current working status	No	1927	74.2
	Yes	670	25.8
Husband education	No education	724	31.9
	Primary	1024	45.1
	Secondary	353	15.6
	Higher	169	7.4

Others^a^ = catholic and traditional religion followers

### Reproductive factors distribution of respondents

Among the total respondents, 1682 (64.80%) of the study participants were not using contraceptive methods. Majority of the study participants, 1257 (48.40%) had their first sex between 15 and 17 years old inclusive. Great proportion of participants, 1156 (44.50%) have married between their 15 and 17 years inclusive (Table 2).

**Table 2:** Reproductive factors distribution of married female youths in Ethiopia, 2016

Variables	Categories	Frequency	Percent
Abortion history	Yes	145	5.6
	No	2452	94.4
Contraceptive usage	Users	915	35.2
	Non-users	1682	64.8
Demand for contraceptive	Unmet need	430	16.5
	Met need	915	35.30
	No demand	1252	48.20
Age at first sex	< 15	524	20.20
	15–17	1257	48.4
	≥ 18	816	31.40
Age at first marriage	< 15	571	22.00
	15–17	1156	44.50
	≥ 18	870	33.50
Husbands desire for children	Both want same	995	43.90
	Husband wants more	447	19.60
	Husband wants fewer	828	36.50

### Behavioral and media related factors of respondents

Out of 2597 respondents, 1891 (72.80% %) have no media exposure and 1922 (74.00%) have not heard family planning messages on mass medias. Among the study participants, 851 (32.80%) and 353 (13.60%) had alcohol drinking and chat chewing history respectively (Table 3).

**Table 3:** Behavioral and media related factors distribution of ever married female youths in Ethiopia, 2016

Variables	Categories	Weighted frequency	Weighted percentage
Alcohol drinking	Yes	851	32.80
	No	1746	67.20
Cigarette smoking	Yes	22	1.00
	No	2575	99.00
Chat chewing	Yes	353	13.60
	No	2244	86.40
Husband drinks alcohol	No	749	70.60
	Yes	311	29.40
Media exposure	Not exposed	1891	72.80
	Exposed	706	27.20
Hearing family planning message on mass-medias	No	1922	74.00
	Yes	675	26.00

### First birth status of the respondents

**Fig. 2:**
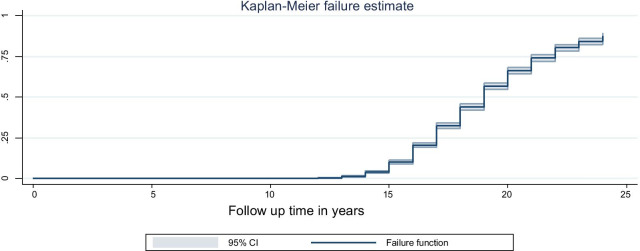
Overall Kaplan–Meier failure curve of ever married female youths in Ethiopia, 2016

From the total of 2597 study participants, 1809 (69.70%) give birth for the first time during the follow up time. Among the respondents who gave birth, 107 (5.90%) give birth before their 15th birth day, 1251 (69.10%) between their 15 and 19 years old inclusive and 451 (24.90%) after 20 years old. About 788 (30.30%) individuals didn’t give birth (they are censored) during the follow up period. The total follow-up time contributed by all study participants was 50,016-person years. The overall median survival time was 18 years (IQR = 17–20). The median survival time varies by respondents’ characteristics. For example, median survival time for urban was 20 years and for rural, it was 18 years. By wealth index, median time for poor, middle and riches were 17.5, 18 and 19.5 respectively. By education, the median age ranges from 17 years for no formal education to 23 years for higher education. The minimum and maximum follow up time for mothers to have first birth was 12 and 24 years respectively (Fig. 2).

### Comparisons of survival functions of different categorical variables

**Fig. 3:**
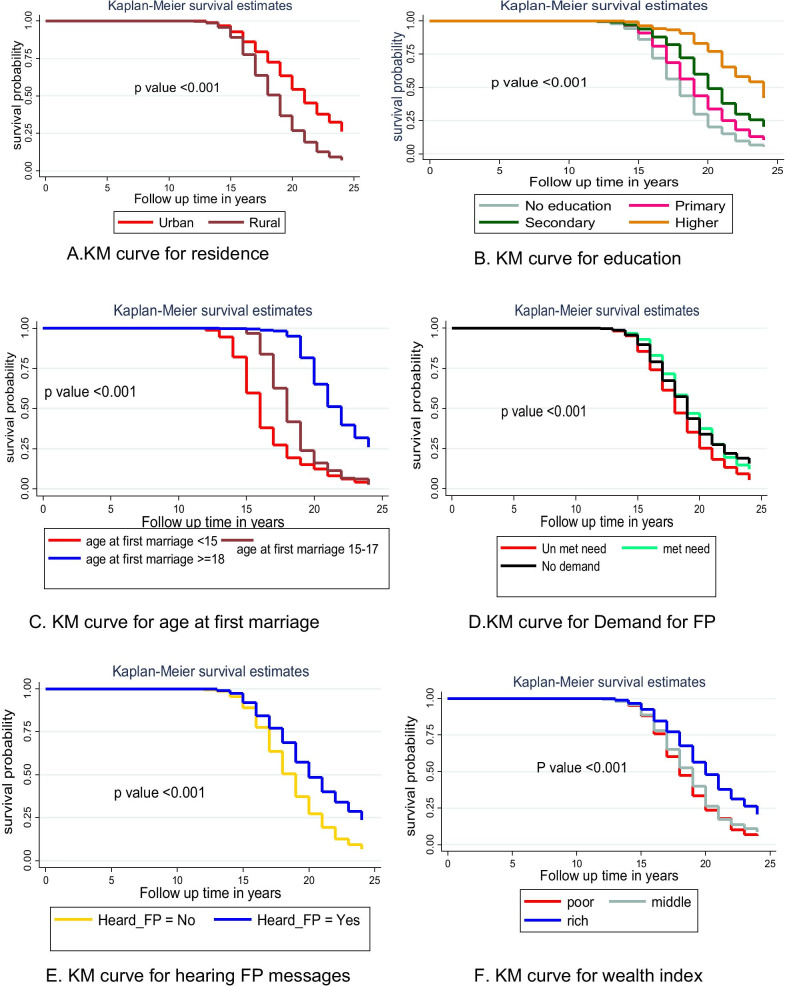
Kaplan–Meier survival curves and log rank tests of ever married female youths by their characteristics in Ethiopia, 2016. *N.B KM* Kaplan–Meier, *FP* family planning

Differences in key variables at base-line among different categories were determined using the Kaplan Meier survival function and the log-rank test. The Kaplan Meier survival function was constructed for different categorical variables. In general, the pattern of the survivorship function lying above another indicated that the group defined by the upper curve had a longer survival than the group defined by the lower curve. For instance, urban residents have longer survival than their rural counter parts at log rank *p* value < 0.001 (Fig. 3a). The significance of the graphically observed difference was assessed by log rank test and it is indicated in the respected.

### Model selection

**Fig. 4:**
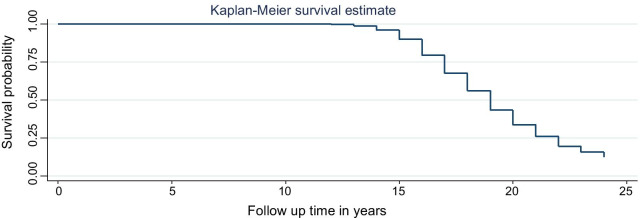
Base line hazard distribution plot to check whether the data has specific distribution or not

The model selection in this study was done based on the distributional assumption of the base line hazard. Based on this assumption, if the baseline hazard had a specific distributional pattern, comparison will be made among parametric survival models. If the base line hazard has no specific distributional pattern, simple Cox regression will be fitted. In this study the baseline hazard had not any distributional pattern. So, simple Cox regression was fitted (Fig. 4).

### Predictors of time to first birth

From thirteen variables which were significant at *p* value < 0.25 in the bivariable analysis (residence, religion, education level, wealth index, working status, husband education, age at first sex, age at first marriage, demand for family planning, decision on marriage, alcohol drinking, media exposure and hearing family planning messages on mass medias), five variables (residence, religion, demand for family planning, age at first sex, and age at first marriage) were significantly associated with the timing of the first birth at *p* < 0.05 with 95% confidence interval (Table 4).

**Table 4:** Bivariable and multivariable Cox regression analysis for determinants of first birth among ever married female youths in Ethiopia, 2016

Variable	Categories	Outcome		CHR (95%CI)	AHR (95% CI)
		Censored	Event		
Residence	Urban	195	178	1	
	Rural	593	1631	2.46 (2.01, 3.02)	1.49 (1.13, 1.97)*
Religion	Orthodox	436	628	**1**	
	Muslim	237	733	1.71 (1.44, 2.03)	1.57 (1.22, 2.02)^**^
	Protestant	106	379	1.50 (1.21, 1.84)	1.73 (1.34, 2.24)**
	Others^a^	9	69	1.91 (1.55, 2.37)	1.35 (.94, 1.92)
Education level	No education	159	714	4.54 (3.38, 6.11)	1.55 (.99, 2.41)
	Primary	428	886	3.02 (2.27, 4.01)	1.26 (.85, 1.87)
	Secondary	144	160	1.58 (1.15, 2.17)	1.03 (.67, 1.57)
	Higher	58	48.72	1	
Wealth index	Poor	285	872	1.75 (1.50, 2.03)	.89 (.76, 1.07)
	Middle	150	371	1.51 (1.27, 1.80)	.81 (.65, 1.00)
	Rich	353	566	1	
Current working status	No	523	1404	1.46 (1.25, 1.70)	1.17 (.96, 1.42)
	Yes	265	405	1	
Husband education	No education	146	578	3.08 (2.35, 4.04)	1.31 (.89, 1.92)
	Primary	254	770	2.44 (1.87, 3.181)	1.22 (.85, 1.75)
	Secondary	128	225	1.53 (1.08, 2.16)	1.14 (.79, 1.66)
	Higher	84	85	1	
Age at first sex	< 15	109	416	5.04 (4.00, 6.34)	1.68 (1.23, 2.29)**
	15–17	345	912	2.78 (2.44, 3.17)	1.54 (1.29, 1.85)^**^
	≥ 18	335	481	**1**	
Age at first marriage	< 15	91	480	8.09 (6.46, 10.16)	6.52 (4.91, 8.64)^**^
	15–17	316	840	3.28 (2.87, 3.75)	2.63 (2.20, 3.141)^**^
	≥ 18	381	489	**1**	
Demand for contraceptives	Met need	260	655	**1**	
	Unmet need	98	332	1.31 (1.07, 1.60)	1.23 (1.00, 1.52) ^*^
	No demand	430	822	.96 (.82,1.12)	.74 (.54,1.17)
Decision on marriage	My self	387	780	**1**	
	Parents	373	945	1.33 (1.15, 1.54)	1.040 (.87, 1.24)
	Other relatives	28	75	1.13 (.81, 1.59)	.79 (.54, 1.17)
Alcohol drinking	Yes	337	514	1	
	No	451	1295	1.41 (1.22, 1.63)	1.23 (.98, 1.55)
Media exposure	Exposed	266	440	1	
	Non-exposed	522	1369	1.53 (1.32, 1.77)	.88 (.71, 1.08)
Hearing FP messages on medias	Yes	266	409	1	
	No	522	1400	1.62 (1.35, 1.95)	1.19 (.95, 1.49)

Bold indicate the signifacant explanatory variabales with thier adjusted hazard ratio and confidence interval

Other ^a^_=_ traditional and catholic

*FP* family planning

^*^Significant at *p* < 0.05 and

^**^Significant at *p* < 0.001

The hazard of first birth among rural residents was increased by 49% (AHR = 1.49, 95% CI 1.13, 1.97) when compared to urban residents keeping others constant.

Religion also affects timing of first birth. The hazard of first birth at earlier age among Muslims was increased by 57% (AHR = 1.57, 95% CI 1.22, 2.02), among protestant it was increased by 73% (AHR = 1.73, 95% CI 1.34, 2.24) when compared to orthodox keeping others constant.

Youths who have started sex < 15 years have 68% increased hazard of first birth (AHR = 1.68,95% CI 1.23, 2.29) and those who started sex between 15 and 17 have 54% increased hazard of first birth (AHR = 1.54, 95% CI 1.29, 1.85) at earlier age than individuals who started sex at age ≥ 18 years keeping others constant.

Youths who have married < 15 years have 6.52 times increased hazard of first birth (AHR = 6.52, 95% CI 4.91, 8.64) and those who have married between 15 and 17 have 2.63 times increased hazard of first birth (AHR = 2.63, 95% CI 2.20, 3.14) at earlier age than individuals who married at age ≥ 18 years keeping others constant.

Having unmet need for family planning increases the hazard of first birth at earlier age by 1.23 times (AHR = 1.23, 95% CI 1.00, 1.52) when compared to those who have met need keeping others constant (Table 4).

Baseline hazard distribution.

## Discussion

This study assessed the time to first birth and its determinants among ever married female youths. The finding showed that the median age at first birth was 18 (IQR = 17–20) years and the determinants of time to first birth among the study participants were residence, religion, age at first sex, age at first marriage, unmet need for family planning, and hearing family planning messages on mass medias.

In this study the median age at first birth was 18 (IQR = 17–20) years. This finding was in line with finding from Swaziland [31], Nigeria [21] and Uganda [32], in which the median age at first birth was 18.22 and 19 and 19.2 respectively. This similarity may be due the reproductive factors similarity in those developing countries. For example, in Uganda, the contraceptive prevalence rate (CPR) among currently married women was 35% [32], the prevalence of unmet need for family planning was 28% [32], the median age at first marriage was 18.7 [32] and the age at first sexual intercourse was 16.9 [32] which is nearly similar to Ethiopian context.

However, our finding was lower than the findings from different countries in which the median age at first birth was 22.6 years in Egypt [33], 21.4 years in Ghana [34], and 20.3 years in Kenya [35]. This difference could be explained by the difference in the contraceptive prevalence rate (CPR), the prevalence of unmet need for family planning, the median age at first marriage and the age at first sexual intercourse. For example, in Egypt CPR among currently married women was 59%, unmet need for family planning was 13%, median age at first marriage was 20.8 years [33]. Whereas in Ethiopia, CPR for currently married women was 36%, unmet need was 22%, median age at first marriage was 17.1 and median age at first sex was 16.6 years [12].

According to our finding, residence was one of the determinants for the median time to first birth. Rural youths have shorter survival than urban. This finding is similar to findings in Nigeria [21], in Swaziland [31] Bangladesh [1, 36], Uganda [37], Gahanna [15], Tanzania [38] and Vietnam [39]. The possible explanation could be, urban women are likely to be educated and likely to be from educated parents which favored urban girls in terms of guidance, motivation and mentoring and would have high modern contraceptive uptake whereas, women in the rural areas may not be well informed about the use of contraceptives and the effects of early child bearing [21, 37, 40].

Age at marriage was also positively associated with age at first childbirth. Female youths who have married early had first child birth earlier than those who have delayed marriage. This finding is in agreement with findings from Nigeria [21] and Bangladesh [1, 36, 41, 42]. Age at marriage has been regarded as one of the proximate determinants of fertility [27]. This may be due to bidirectional relationship between age at first marriage and educational attainment as delaying marriage might give room for better education [21]. This is because, education is one of the major factors affecting age at marriage [15].

This study also shows that age at first sexual intercourse has a significant effect on age at first birth. A woman who starts having sexual intercourse at earlier age have the first child at an earlier age compared to a woman who started sex lately. This is in line with findings from Uganda [37] and Swaziland [31]. The possible explanation for this finding could be, the modern contraceptive uptake among early sexual initiators is low compared to late initiators [40].

This study also shows a difference in age at first births along religious affiliations. Among religious affiliations, being Protestant and being Muslim affiliated were found to shorten the survival time of first birth as compared to Orthodox. Regarding to being Muslims, similar findings were documented in Bangladesh [41] and Nigeria [21] which shows that women in Islamic religion had a tendency of early first birth than women in other religions. This may be due to the likely hood of muslins to marry at age less than 15 years [41, 42]. In this study, among women who have married at age less than 15 years, about 42% were Muslims. Regarding to being protestant, there were supportive evidence in Ghana which shows the liberal attitude of women towards sexual activity increase the likely hood of women’s premarital sexual intercourse [43]. The difference regarding to religion may also be explained by attitudes, norms and beliefs regarding birth control and the value of children among different religions [15].

Another determinant of timing of first birth was unmet need for family planning. Having unmet need for family planning shortens the timing of first birth when compared to those who have met need. This finding is in line with findings from Nigeria [21, 44] and Bangladesh [41, 45] which report that women who had ever used anything to prevent pregnancy have delayed first birth than those who never used. This could be explained by the fact that those who used something to prevent pregnancy were in control of their fertility and postpone child bearing.

The findings of this study implies that future researchers, policy makers and program managers should focus on the unmet need, age at marriage, age at first sex and the contraceptive uses and their associated factors among of rural female youths to prevent early child bearing and its societal, health and demographic consequences.

### Strength and limitation of the research

The main strength of this research is using nationally representative data and it is generalizable to all Ethiopian married youths. But since the source of the data was self-report, the accuracy of the data could be affected by recall bias. Using secondary data limit the researcher to measure all possible predictors like peer related and cultural related factors.

## Conclusion

In this study, the median age at first birth was 18 years. This show, about 50% of study participants give birth for the first time before their 18th birth day. This age is the ideal age for schooling and to do other personal development activities. Therefore giving birth before 18 year will limit female youths from attending school and performing personal development activities in addition to health and demographic consequences of early child bearing. This finding is comparable with developing countries and lower than the global and developed countries. The timing of first birth in Ethiopia was mainly influenced by socio-demographic and reproductive related factors.

## Recommendations

### For the government of Ethiopia

- Continuing enforcing the legal age of marriage

### For ministry of health

- Expanding youth friendly service for all communities maintaining the quality of the services to reduce unmet need for family planning.
- Better to strictly implementing the national adolescents and youths sexual and reproductive health strategies.

### For ministry of education

- Designing curriculum consisting comprehensive sexual health education in schools to help adolescents and youths delaying sexual initiation and empowering them to delay age of marriage.

### For researchers

- Better to do prospective follow up study to assure the cause and effect or temporal relationship between outcome variable and explanatory variables.

## Data Availability

The data for this study was obtained from the 2016 Ethiopian demographic and health survey and it is available freely through (https://www.dhsprogram.com).

## Abbreviations

AHR: Adjusted hazard ratio
CHR: Crude hazard ratio
CI: Confidence interval
DHS: Demographic health survey
EDHS: Ethiopian demographic health survey
HR: Hazard ratio
IQR: Inter quartile range
VIF: Variance inflation factors
PH: Proportional hazard
IPH: Institute of public health
